# Assessment of Polymer Atmospheric Correction Algorithm for Hyperspectral Remote Sensing Imagery over Coastal Waters

**DOI:** 10.3390/s21124125

**Published:** 2021-06-16

**Authors:** Mariana A. Soppa, Brenner Silva, François Steinmetz, Darryl Keith, Daniel Scheffler, Niklas Bohn, Astrid Bracher

**Affiliations:** 1 Alfred Wegener Institute, Klußmannstr. 3d, D-27570 Bremerhaven, Germany; bsilva@awi.de (B.S.); abracher@awi.de (A.B.); 2 HYGEOS, Euratechnologies, 165 Avenue de Bretagne, 59000 Lille, France; fs@hygeos.com; 3 Atlantic Coastal Environmental Sciences Division, United States Environmental Protection Agency, Narragansett, RI 02882, USA; keith.darryl@epa.gov; 4 Helmholtz Center Potsdam GFZ German Research Center for Geosciences, Remote Sensing and Geoinformatics, Telegrafenberg, D-14473 Potsdam, Germany; daniel.scheffler@gfz-potsdam.de (D.S.); niklas.bohn@gfz-potsdam.de (N.B.); 5 Institute of Environmental Physics, University of Bremen, D-28334 Bremen, Germany

**Keywords:** water colour, ocean colour, water reflectance, EnMAP

## Abstract

Spaceborne imaging spectroscopy, also called hyperspectral remote sensing, has shown huge potential to improve current water colour retrievals and, thereby, the monitoring of inland and coastal water ecosystems. However, the quality of water colour retrievals strongly depends on successful removal of the atmospheric/surface contributions to the radiance measured by satellite sensors. Atmospheric correction (AC) algorithms are specially designed to handle these effects, but are challenged by the hundreds of narrow spectral bands obtained by hyperspectral sensors. In this paper, we investigate the performance of Polymer AC for hyperspectral remote sensing over coastal waters. Polymer is, in nature, a hyperspectral algorithm that has been mostly applied to multispectral satellite data to date. Polymer was applied to data from the Hyperspectral Imager for the Coastal Ocean (HICO), validated against in situ multispectral (AERONET-OC) and hyperspectral radiometric measurements, and its performance was compared against that of the hyperspectral version of NASA’s standard AC algorithm, L2gen. The match-up analysis demonstrated very good performance of Polymer in the green spectral region. The mean absolute percentage difference across all the visible bands varied between 16% (green spectral region) and 66% (red spectral region). Compared with L2gen, Polymer remote sensing reflectances presented lower uncertainties, greater data coverage, and higher spectral similarity to in situ measurements. These results demonstrate the potential of Polymer to perform AC on hyperspectral satellite data over coastal waters, thus supporting its application in current and future hyperspectral satellite missions.

## 1. Introduction

The vast information provided by hyperspectral sensors allows us to comprehensively explore the upwelling spectral radiance distribution from water and to improve the retrieval of geo-biophysical parameters. The wide range of applications includes the detection of phytoplankton types and harmful algal blooms, benthic habitat mapping, and phytoplankton fluorescence retrievals. Past, current, and future hyperspectral sensors with potential application in water colour studies include Hyperion (2000–2017), HICO (2009–2014), SCIAMACHY (2002–2012), CHRIS-PROBA (2001–), OMI (2004–), GOME-2 (2006–, 2012– and 2018–), TROPOMI (2017–), DESIS (2019–), PRISMA (2019–), HISUI (2019–), EnMAP (2022–), OCI (PACE mission, 2023–), PRISMA SG (2025–), SHALOM (2025–), SBG (2027–), and CHIME (2029–).

Nearly all water colour algorithms require atmospheric correction (AC) of radiance (or reflectance) backscattered by the water (water-leaving radiance, L_*W*_; water-leaving reflectance, ρ_*w*_) as input. L_*W*_ carries the information of the water optical properties and constitutes only a small fraction of the radiance measured by the sensor. Thus, AC is key to obtaining accurate retrievals over water using spaceborne remote sensing. There exist several AC algorithms; however, most of them have been designed and applied to multispectral sensors, as hyperspectral spaceborne data appropriate for water colour research and application are limited. To date, only HICO, operated on the International Space Station (ISS), and OCI sensors have been specifically designed for water colour applications, such as signal-to-noise ratio (SNR), band location, and spatial/temporal resolution. The other sensors are primarily designed for atmospheric and land-use applications. However, the recent launch of hyperspectral sensors with characteristics suitable for water colour research (e.g., DESIS and PRISMA) and the new ones set to launch in the coming years (e.g., EnMAP, OCI, SBG, SHALOM, and CHIME) will require AC algorithms that can cope with hundreds of narrow spectral bands and atmospheric/surface effects. The main difficulties faced by AC algorithms over water bodies are handling properly absorbing aerosols, trace gases and water vapor, whitecaps, sun glint, highly complex waters, and adjacency effects, as has been highlighted in the thorough review of AC in hyperspectral remote sensing by [1].

To date, two AC algorithms have been specifically designed for water colour remote sensing, and are freely available for scientific use, and able to handle hyperspectral satellite data—the POLYnomial-based algorithm applied to MERIS (Polymer) [2,3] and NASA’s standard AC algorithm (L2gen) [4,5,6]. In the framework of the PACE mission, a hyperspectral version of the L2gen was developed for application to HICO data, and was validated against in situ measurements, showing very good agreement [6]. Major improvements in the algorithm comprise the compensation for water vapor absorption, correction of Rayleigh and aerosol scattering, and the derivation of new vicarious gains [6]. Polymer is a spectral matching algorithm which uses the full spectra available, together with an iterative optimisation scheme, to separate the radiometric contribution of water from the atmospheric and surface contributions. Evaluation of Polymer applied to multispectral data showed improved performance over other existing AC methods and increased data coverage, due to its ability to handle thin clouds and sun glint [2,7,8,9,10,11,12]. Another strength of Polymer, compared with other ocean colour AC algorithms, is that it can be applied to several sensors, thus maximising the consistency between products [7]. This is a key point, as long time-series of ocean colour products with high temporal and spatial resolution are only accessible through the synergy of different sensors. However, an evaluation of Polymer applied to hyperspectral satellite data is still missing.

In support of the Environmental Mapping and Analysis Program (EnMAP) scientific preparation program, we investigate the performance of Polymer on hyperspectral remote sensing data over coastal waters. EnMAP is a German hyperspectral satellite mission to be launched in 2022, which aims to characterise the Earth’s terrestrial and marine ecosystems at the global scale [13]. The mission will offer great advantages, in terms of sensor characteristics, compared with existing ocean colour sensors: 242 bands from 420–2450 nm at 6.5 nm in the visible and near-infrared (VNIR) and 10 nm in the short-infrared (SWIR), SNR ≥ 400:1 in the visible (VIS), spatial resolution of 30 m, and a revisit period of 4 days with 30^∘^ across-track viewing. We tested Polymer using HICO data acquired at seven coastal areas encompassing a wide range of optical characteristics, and where multispectral or hyperspectral in situ radiometric measurements are available. In addition, we compared its performance against that of L2gen. Finally, we implemented Polymer in the EnMAP-Box, an open source plugin for QGIS for the processing and analysis of EnMAP data, and tested the application using simulated EnMAP imagery.

## 2. Materials and Methods

### 2.1. Atmospheric Correction Algorithms

The Polymer algorithm can be divided into a three-step process: pre-correction of the top of atmosphere radiance (L_*TOA*_), spectral matching of the atmospheric and water models, and retrieval of the water-leaving reflectance. First, L_*TOA*_ measured by the sensor is converted into reflectance (ρ_*TOA*_). After correction for the gaseous transmittance estimated for O_3_ using ancillary data, and for NO_2_ with climatology data, using the same spectral absorption coefficients as L2gen, ρ_*TOA*_ can be described as the sum of the reflectance due to scattering by air molecules (Rayleigh scattering, ρ_*mol*_); sun glint reflectance (ρ_*gli*_ with transmission factor *T*); aerosol reflectance (ρ_*aer*_); the coupling between sun glint, molecules, and aerosols (ρ_*coup*_); and the water-leaving reflectance just above the surface ($\rho_{w}^{+}$) with direct and diffuse atmospheric transmission (*t*):

(1) $$\rho_{TOA}\left(\lambda \right)=t_{oz}\left(\lambda \right)t_{NO2}\left(\lambda \right)\left[\rho_{mol}\left(\lambda \right)+T\rho_{gli}+\rho_{aer}\left(\lambda \right)+\rho_{coup}\left(\lambda \right)+t\left(\lambda \right)\rho_{w}^{+}\left(\lambda \right)\right].$$

The ρ_*TOA*_ undergoes initial corrections for gaseous absorption, Rayleigh scattering, and a sun glint pre-correction, resulting in a pre-corrected reflectance ($\rho^{\prime }$). This $\rho^{\prime }$ contains a residual sun glint (Δρ_*gli*_), the aerosol reflectance, the coupling terms, and the ocean water-leaving reflectance:

(2) $$\rho^{\prime }\left(\lambda \right)=\Delta \rho_{gli}\left(\lambda \right)+\rho_{aer}\left(\lambda \right)+\rho_{coup}\left(\lambda \right)+t\left(\lambda \right)\rho_{w}^{+}\left(\lambda \right)].$$

Subsequently, $\rho^{\prime }$ can be split into the atmospheric and sun glint (ρ_*ag*_) and water components (*t*.$\rho_{w}^{+}$):

(3) $$\rho^{\prime }\left(\lambda \right)=\rho_{ag}\left(\lambda \right)+t\left(\lambda \right)\rho_{w}^{+}\left(\lambda \right)],$$

where ρ_*ag*_ represents

(4) $$\rho_{ag}\left(\lambda \right)=\Delta \rho_{gli}\left(\lambda \right)+\rho_{aer}\left(\lambda \right)+\rho_{coup}\left(\lambda \right).$$

The second step (which comprises the core of Polymer) is spectral matching. The parameters of the atmospheric and water-leaving reflectance models are optimized, in order to obtain the best spectral fit of $\rho^{\prime }$. The atmospheric model that represents ρ_*ag*_ is a linear combination of three terms depending on the wavelength, which account for spectrally flat components and the beam attenuation due to Rayleigh scattering (first term), the aerosol signal with a spectral dependency representing an aerosol fine mode (second term), and the couplings between flat components and the Rayleigh scattering (third term):

(5) $$\rho_{ag}\left(\lambda \right)=T\left(\lambda \right)c_{0}+c_{1}\lambda^{-1}+c_{2}\rho_{mol}\left(\lambda \right).$$

The water-leaving reflectance model is based on the bio-optical model of [14], developed for both case 1 and case 2 waters. Modifications of the original water-leaving reflectance model have been described in [3]; for instance, the model described in [14] also includes a dependence on the coloured dissolved organic matter absorption variability, which is neglected in Polymer, as it introduces instability into the algorithm. In Polymer, the water-leaving reflectance model depends on the wavelength, the observation geometry, and two free parameters—chlorophyll-a concentration (*chl*) and a coefficient *f_b_* that scales the backscattering coefficient of particles:

(6) $$\rho_{w}^{+}\left(\lambda \right)=f\left(chl,f_{b}\right).$$

The spectral matching is performed using 50 bands of HICO between 410 nm and 776 nm, excluding those affected by gaseous absorption bands (e.g., those between 668 and 736 nm which are affected by oxygen-B, ozone, and water vapor absorption). Bands greater than 800 nm present spectral calibration problems in HICO [6], and should be avoided. Finally, when the optimisation of the free parameters (*c*_0_, *c*_1_, *c*_2_, *chl*, and *f_b_*) is achieved, the water-leaving reflectance can be estimated by:

(7) $$\rho_{w}^{+}\left(\lambda \right)=\frac{\rho^{\prime }\left(\lambda \right)-T\left(\lambda \right)c_{0}+c_{1}\lambda^{-1}+c_{2}\rho_{mol}\left(\lambda \right)}{t(\lambda )}.$$

The $\rho_{w}^{+}$ is further normalised to the nominal wavelength and for the water-leaving reflectance bidirectional effects (BRDF), as described in [3], such that the radiometric output of Polymer is the fully normalised water-leaving reflectance spectrum corrected for bidirectional effects. Detailed information on the pre-correction (e.g., estimation of the gaseous transmittance, Rayleigh scattering, and pre-glint correction), spectral matching, and the atmospheric model has been presented in [2,3]; information on the water-leaving reflectance model can be found in [14]; and its modifications, water-leaving reflectance normalisation, and BRDF correction have been detailed in [3]. The vicarious calibration gains of HICO, derived by [6], are also accounted for in Polymer and applied to the level 1B input product.

L2gen is based on the NASA heritage AC algorithm—a standard atmospheric correction scheme, in which the aerosol signal is estimated in the red, near infrared (NIR), or short wave infrared (SWIR) bands and extrapolated to the visible bands. The initially developed L2gen [4] assumed that the water-leaving reflectance is negligible in the NIR region, leading to significant errors in highly productive and optically complex waters, where this assumption does not hold. The study in [15] revised the algorithm by including a bio-optical model with an iteration scheme, in order to estimate the water-leaving reflectance in the NIR. L2gen was further adapted and improved by [6], supporting its application to hyperspectral sensors. It includes the ATmospheric REMoval code to estimate and correct for the absorption of seven gases (water vapor, ozone, oxygen, nitrous oxide, carbon monoxide, methane, and carbon dioxide) and extends the multispectral Rayleigh and aerosol correction to the hyperspectral domain. The radiometric output of L2gen is the remote sensing reflectance (Rrs) spectrum, including bidirectional reflectance correction. To compare the radiometric output of L2gen and Polymer, the normalised water-leaving reflectance of Polymer can be converted to Rrs by dividing its value by π [1].

### 2.2. In Situ Radiometric Data

**Figure 1:**
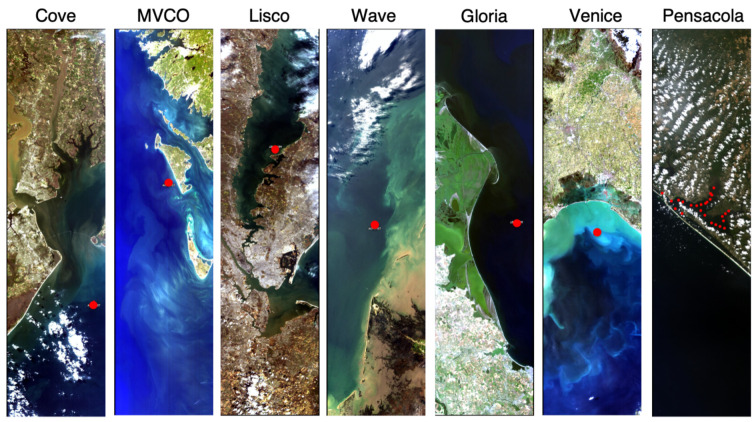
True-colour image composites of HICO Level 1B data at AERONET-OC sites and Pensacola Bay. Red circles show the location of the AERONET-OC and sampling stations.

The multispectral radiometric measurements were obtained from the AERosol Robotic NETwork—Ocean Colour (AERONET-OC) at six coastal sites (Figure 1): COVE_SEAPRISM (Cove), MVCO, LISCO, WaveCIS_site_CSI_6 (Wave), Gloria, and Venice. Quality-controlled (level 2.0) normalised water-leaving radiances corrected for bidirectional effects [16] were converted to Rrs by dividing by *F*_0_, the extraterrestrial solar irradiance at the mean Earth–Sun distance [17]. The data set was available in five or six spectral bands in the VIS range, which varied or changed slightly with location and/or year (Table 1) and are here named as 412, 441, 490, 530, 551, and 668 nm. These spectral bands were compared to HICO bands at 410, 444, 490, 530, 553, and 668 nm.

The hyperspectral data set was shared by [18], and is composed of quality-controlled Rrs measurements at 1 nm spectral resolution for the 400–735 nm range at 25 stations at Pensacola Bay ($30^{\circ }42^{\prime }$ N, $87^{\circ }22^{\prime }$ W) on 2 June 2011. Pensacola Bay is a shallow coastal estuary system connected to the Gulf of Mexico, which is largely influenced by river runoff [19]. The measurements were collected by an above-surface radiometry system (HyperSAS, Satlantic Inc., Halifax, Nova Scotia); details on the measurements and data processing can be found in [20]. HICO Rrs data for the 410–719 nm range were paired to the same wavelengths of HyperSAS Rrs data for comparison. HICO’s spectral response function was not accounted for because the data were available as reflectance and the spectral convolution should be performed on the radiance space [21].

### 2.3. Satellite Data and Validation

HICO [22,23] level 1B and level 2 images were obtained for the seven sites from NASA’s ocean colour website [24]. The level 1B data set was processed with Polymer version 4.11, using the default settings. The Polymer-normalised water-leaving reflectance was converted to Rrs by dividing by π [1]. The HICO level 2 data set corresponds to Rrs products processed with the hyperspectral-L2gen [6], and was used for comparison.

To derive high-quality match-ups, a strict quality control was applied, including a time window of ±3 h between in situ measurements and HICO overflights and a spatial homogeneity check over a 3 × 3 pixel box centered on the location of the in situ data. All nine pixels were checked, regarding the quality control flags provided by the AC algorithms. For Polymer, the flags applied were: ‘land’, ‘cloud base’, ‘external mask’, ‘high air mass’, ‘thick aerosol’, ‘exception’, ‘level 1 invalid’, and ‘negative bb’. The L2gen flags assigned were ‘land’, ‘atmfail’, ‘straylight, ‘hilt’, and ‘cldice’. Match-ups are regarded as valid if at least 50% of the pixels remained after the assignment of flags and if the coefficient of variation (ratio of the standard deviation to the median value) of satellite Rrs (410, 444, and 490 nm) in the pixel box was smaller than 0.15. The median values of the remaining pixels were then used for statistical analyses. If more than one in situ measurement was collocated to the same satellite data, the median value was used for the match-up exercise.

To compare the satellite products and in situ measurements, three performance metrics were selected, following [25,26]: the mean absolute percentage difference (MAPD, as an index for dispersion), the mean percentage difference (MPD, as an index for bias), and the spectral angle (SA, as an index for spectral similarity). The MAPD and MPD metrics are defined as:

(8) $$MAPD=\frac{\sum_{i=1}^{N}100\left|\frac{y_{i}-x_{i}}{x_{i}}\right|}{N},$$

(9) $$MPD=\frac{\sum_{i=1}^{N}100\left(\frac{y_{i}-x_{i}}{x_{i}}\right)}{N},$$

where *x* and *y* are the in situ and satellite-estimated Rrs data, respectively, and *N* is the total number of samples.

The SA (θ, degrees) was used to investigate the agreement between in situ and satellite-derived Rrs spectra. The smaller the spectral angle, the higher the similarity in the spectral shape. The spectral angle θ is defined as [27]:

(10) $$\theta \left(x,y\right)=\cos^{-1}\left(\frac{\sum_{i=1}^{n}\left(x_{i}\cdot y_{i}\right)}{\sqrt{\sum_{i=1}^{n}x_{i}^{2}}\sqrt{\sum_{i=1}^{n}y_{i}^{2}}}\right),$$

where *x* and *y* are the in situ and satellite Rrs data, respectively, and *n* is the number of spectral bands.

## 3. Results and Discussion

**Figure 2:**
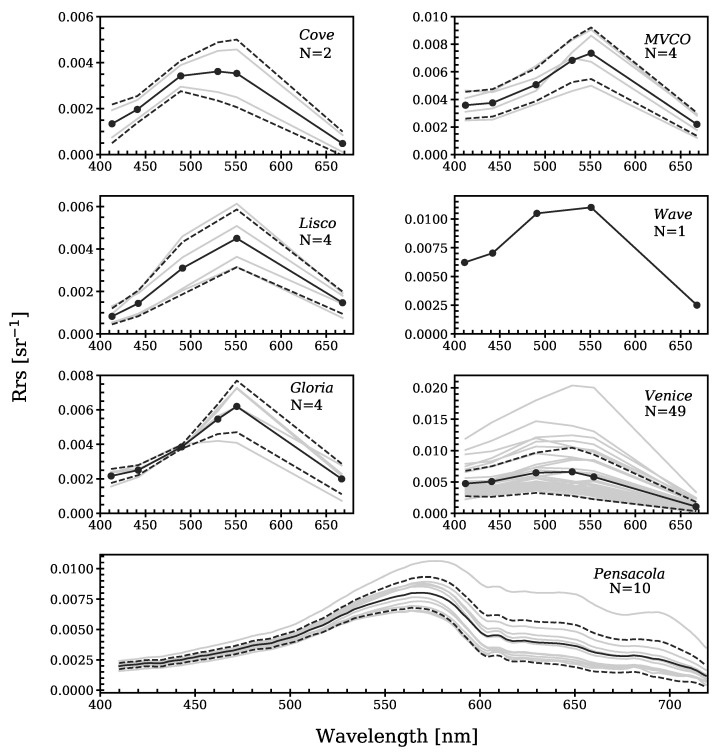
In situ Rrs spectra from AERONET-OC sites and Pensacola Bay (match-ups). The continuous black lines indicate mean values, the dashed lines indicate ±1 standard deviation and the grey lines are all matched data with Polymer and L2gen. N represents the number of match-up spectra at each region.

The mean match-up in situ Rrs spectra of the investigated sites exhibited two major spectral shapes (Figure 2). At the MVCO, Lisco, Gloria, and Pensacola sites, the mean spectra presented a gradual increase in the Rrs until the peak around 550–570 nm, where they started to decrease towards the longer wavelengths. At the Cove, Wave, and Venice sites, this initial increase in the reflectance was less sharp and there was no clear peak at 550 nm; however, a broad region of high reflectance between 490 and 560 nm was observed. The higher number of match-ups at the Venice site, compared with the other regions, allowed us to observe the significant variability in the spectral shape caused by the wide range of bio-optical conditions, due to the location of the tower between open and coastal waters [28].

### 3.1. Match-Up Analysis

**Figure 3:**
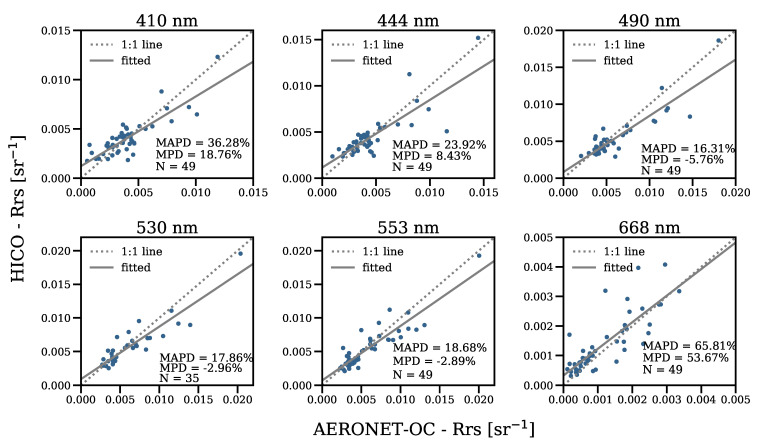
Scatterplots of the AERONET-OC Rrs versus Polymer-HICO Rrs. The two match-ups with both flags ‘case-2’ and ‘inconsistency’ raised were excluded before the statistics were computed.

Statistical analysis of the match-ups revealed that Polymer retrieved Rrs with uncertainties (MAPD) in the range of 16.31% to 65.81%, with bias (MPD) between −2.89% and 53.67%, where the best performance was in the green bands at 530 and 553 nm, and the lowest was in the 668 nm red band (Figure 3). Comparison to in situ measurements did not show systematic under-/over-estimation of the Rrs, but suggested over-correction of the atmospheric/surface effects between 490 and 553 nm and under-correction at 410, 444, and 668 nm. In the blue band (410 nm), the ratio of water-leaving reflectance to top-of-atmosphere reflectance is lower and there is higher uncertainty in the aerosol correction, due to increased Rayleigh scattering at blue wavelengths; in the red band (668 nm), the water absorption increases towards the longer wavelengths, such that the Rrs signal becomes very small, thus increasing the relative errors (Figure 2 and Figure 3). These issues make AC more challenging at these spectral regions, compared with other bands. Further, we did not observe a dependence of the differences between HICO-Rrs and in situ-Rrs per band on the aerosol optical thickness, wind, or solar zenith angle, suggesting that the hyperspectral Polymer is robust with respect to illumination and environmental conditions.

Although no other evaluation of Polymer applied to hyperspectral satellite data has been carried out, we can compare our results of the Rrs match-up analysis to the literature on the performance of Polymer applied to multispectral data in optically complex waters. The uncertainties observed here were lower or close to those reported in recent studies evaluating OLCI (Ocean and Land Colour Instrument on board Sentinel-3 satellites) Polymer radiometric retrievals in inland and coastal waters [12,29]. For instance, ref. [29] reported MAPD ranging from 22 to 43% in the blue bands (412–490 nm) for Estonian inland and Baltic Sea coastal waters; our results showed differences between 16 and 36%. Except for the red band 668 nm, the biases were also much lower than those reported by [12] for the coastal waters of British Columbia and Southeast Alaska.

The EnMAP mission, as dedicated ocean colour missions, targets radiometric uncertainty within 5%; this requirement was not achieved with Polymer or L2gen. However, the uncertainties presented here include not only those associated with AC algorithms, but also with the HICO sensor (e.g., calibration), in situ measurements (e.g., instrument calibration), and match-ups (e.g., spatial and temporal mismatches). Studies on HICO have confirmed the potential of hyperspectral technology for water colour research [18,30,31,32,33], but the sensor presented issues with radiometric and spectral calibration and geolocation accuracy, as HICO was aboard the ISS. These issues have been improved considerably over the years; in particular, through the new vicarious calibration gains derived by [6]. Regarding the in situ radiometers, [28] estimated a 4.5–8% uncertainty in the normalised water-leaving radiances at Venice AERONET-OC. Finally, we attempted to minimise the uncertainty in the match-ups by following strict match-up criteria and applying the recommended quality control flags. Following a very similar match-up protocol, [34] showed that reducing the temporal threshold at the Venice site from 3.5 h to 1 h had little influence on the uncertainties (2% at most); our data set comprised data mostly from the Venice AERONET-OC.

**Figure 4:**
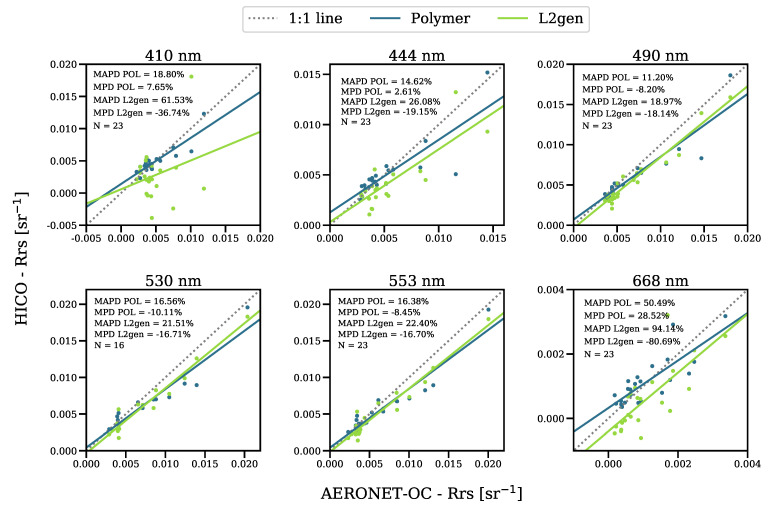
Scatterplots of the AERONET-OC-Rrs versus Polymer (blue) and L2gen (salmon) HICO-Rrs at Venice AERONET-OC; coincident match-ups.

When compared with L2gen, Polymer exhibited lower uncertainties and bias (Figure 4), and provided greater data coverage. For example, the Polymer match-up data set at the Venice site consisted of 35 match-ups, compared with 26 for L2gen. The largest differences between Polymer and L2gen were observed at the bands with weakest Rrs signals; that is, at 410 nm and 688 nm. L2gen also showed systematic underestimation, as has been reported in [6,35], with negative retrievals at 410 nm and 668 nm. Negative retrievals in L2gen may result from uncertainties in the estimation of the aerosol contribution and extrapolation to the shorter wavelengths or adjacency effects [6,15]. The dependence of L2gen on the aerosol models is absent in Polymer, as the algorithm does not use a specific aerosol model but, instead, a linear combination of terms that accounts for the aerosol contribution to the atmospheric reflectance. The AC in Polymer relies on the full visible spectrum and is coupled with a water-leaving reflectance model. This prevents, by construction, strongly negative retrievals at any wavelength.

**Figure 5:**
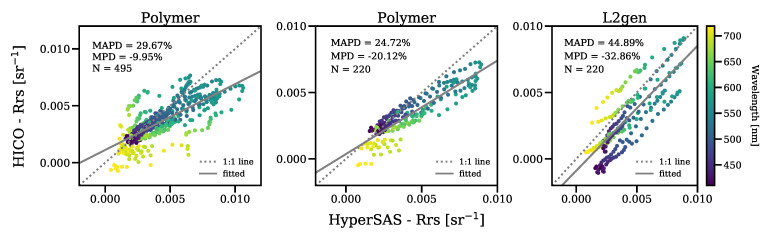
Scatterplots of HyperSAS-Rrs versus HICO-Rrs for the 410–719 nm spectral range: All Polymer match-ups (**left**), and Polymer (**centre**) and L2gen (**right**) coincident match-ups.

The comparison of Polymer Rrs to in situ hyperspectral Rrs (HyperSAS) showed good agreement between these two data sets (Figure 5). Moreover, following our observations in the multispectral analysis, Polymer provided greater data coverage and lower errors than L2gen. In the hyperspectral match-up, we observed that Polymer retrieved a few unrealistic negative values, which occurred in the 719 nm band, likely caused by the influence of the 725 nm water vapor band. As this band was not available for the AERONET-OC sites, these negative Rrs were absent in the multispectral analysis. In L2gen, negative Rrs values were observed at bands ≤456 nm and, in addition, the data points in the L2gen plot show a structure that could not explained, based on our small hyperspectral data set. The 220 data points correspond to only four coincident HyperSAS-Polymer-L2gen stations (Figure 5).

Differences between the spectral resolutions and sampling intervals of HICO and HyperSAS may also have contributed to the observed uncertainties. HICO measured with a 3.2 nm spectral resolution and 1.9 nm spectral sampling, but three bands were binned on board to increase the SNR, resulting in spectral sampling of 5.7 nm and a full-width half-maximum spectral resolution of 10 nm in the spectral range ≤745 nm [36]. HyperSAS instruments have a spectral sampling of 3.3 nm and a spectral width of about 10 nm. The data used here were provided at 1 nm intervals.

#### Quality Flags

**Figure 6:**
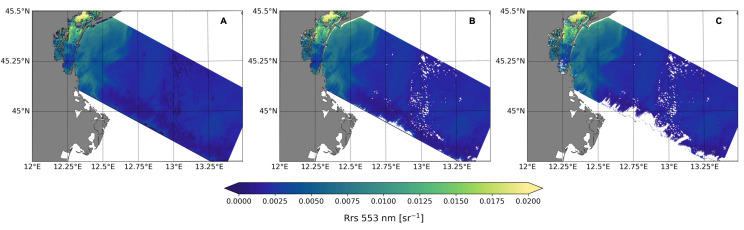
Comparison of the spatial distribution of HICO-Rrs at 553 nm in the northern Adriatic Sea on 26 September 2010: (**A**) no flags; (**B**) recommended flags; and (**C**) recommended flags plus ‘case-2’ and ‘inconsistency’ flags raised together.

Despite applying the recommended flags for Polymer processing, two match-ups with satellite Rrs values close to zero at all wavelengths stood out: one at the Gloria site and one at the Wave AERONET-OC site. These match-ups were excluded from the multispectral match-up data set before computing the statistics; however, we further looked at these unusual data points and found that, in both cases, the flags ‘case-2’ and ‘inconsistency’ were raised. Normally these flags do not invalidate the results; particularly ‘case-2’, which is raised when the optimisation uses initial values adjusted for ‘case 2’ water areas/pixels [37]. The inconsistency flag is raised, if at any band, ρ_atm_ or t*$\rho_{w}^{+}$ exceeds the ρ_TOA_ corrected for Rayleigh scattering [37]. As these were the only cases in the multispectral match-up data set with this flag combination, we searched in the hyperspectral match-up data set for similar cases. There were three match-ups with both flags (i.e., ‘case-2’ and ‘inconsistency’) raised, but the values of Rrs were in the range of the other match-ups. Excluding the hyperspectral match-ups with both ‘case-2’ and ‘inconsistency’ flags raised reduced the MAPD from 29.67% to 27.54%, but the MPD increased from −9.95% to 19.24%, as most of the data removed were overestimating Rrs. Moreover, it removed the variability inherent in the study site. However, future studies should gauge the extent to which these flags are important to filter out low-quality retrievals. The influence of this additional combination of flags on satellite data coverage is illustrated in Figure 6 for the northern Adriatic Sea: more pixels were flagged in the southern limit of the scene when the recommended flags plus ‘case-2’ and ‘inconsistency’ flags were raised together.

### 3.2. Spectral Similarity Analysis

**Figure 7:**
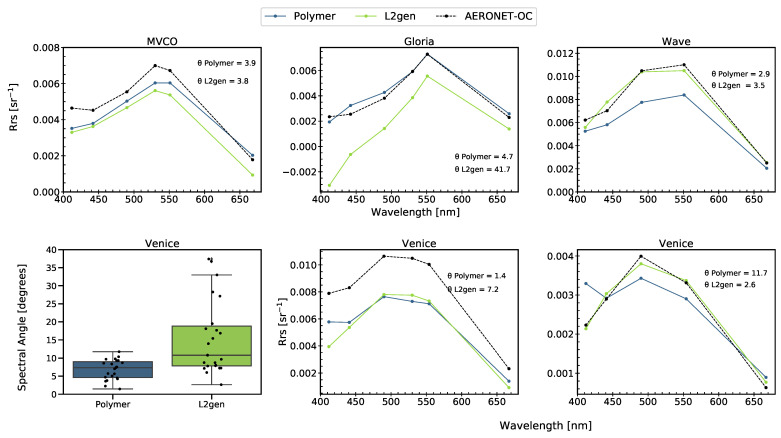
Top: Spectra of coincident match-ups of HICO-Polymer, HICO-L2gen, and AERONET-OC at MVCO, Gloria, and Wave sites (N = 1). Bottom left: boxplots of spectral angle for each AC algorithm at the AERONET-OC Venice site (N = 22). Center lines show the medians, box limits indicate the 25th and 75th percentiles, whiskers extend 1.5 times the interquartile range, and the spectral angle data are represented by data points on top of the boxes. (**Bottom center** and **right**): spectra of coincident match-ups of HICO-Polymer and HICO-L2gen at AERONET-OC Venice for the lowest and highest Polymer spectral angles.

Investigation of the spectral similarity provides additional information about the effectiveness of the algorithms. Both algorithms showed good consistency across the spectra, compared to the in situ multispectral data set (Figure 7), but the performance of Polymer was, on average, better than that of L2gen. For example, the median spectral angle of Polymer at the Venice site was $7.35^{\circ }$, while that of L2gen was $10.78^{\circ }$ (Figure 7, boxplot). These values were similar to those reported by [9], who compared the performance of different AC algorithms applied to OLCI data in optically complex waters on the coast of France; they reported SA values of $7.29^{\circ }$ for Polymer and $14.60^{\circ }$ for L2gen.

**Figure 8:**
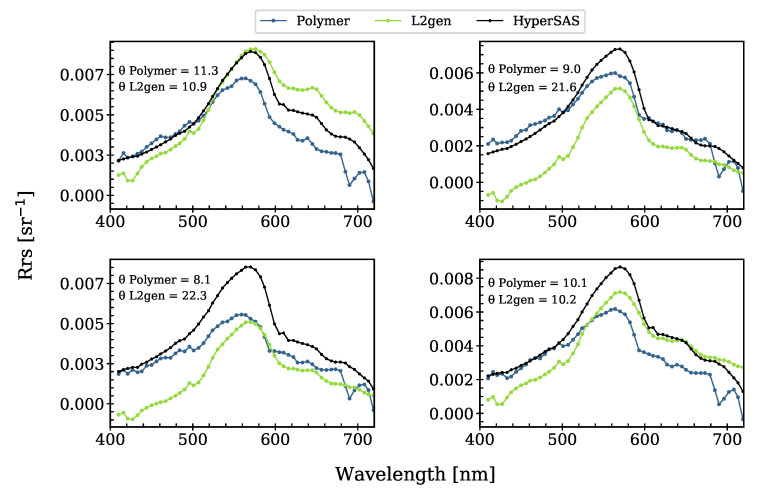
Spectra of coincident match-ups of HICO-Polymer, HICO-L2gen, and HyperSAS for the 410–719 nm spectral range (N = 4).

Looking at the hyperspectral data set (Pensacola Bay), the spectral shape of the L2gen-Rrs spectra was more similar to the in situ HyperSAS spectra than those of Polymer, especially at the peak around 570 nm (Figure 8). L2gen seemed to also better compensate for the spectral features of gaseous absorption (e.g., at 690 nm and 719 nm, caused by the absorption by oxygen-B and water vapor, respectively), as Polymer corrects only for O_3_ and for NO_2_ gaseous absorption. Nevertheless, Polymer performed better in the blue–green spectral region and, overall, the magnitude of its Rrs spectra was closer to the in situ spectra than L2gen-Rrs. This influenced the spectral angle, leading to smaller SA values for Polymer than for L2gen. HICO instrument artifacts were also visible, as slight peaks, at 416 nm and 496 nm.

### 3.3. EnMAP-Box

The Polymer algorithm is currently being integrated into the EnMAP processing tool (EnPT, [38]) of the EnMAP-Box [39], using the wrapper module ACwater. The EnMAP-Box is a software toolbox for processing hyperspectral data, used as a plugin of the QGIS [40] software, and provides the interface to the processing suites developed for imaging spectroscopy. EnPT is a pre-processing chain to process EnMAP data from Level 1B to Level 2A, which can be used as a standalone Python package or within the EnMAP-Box. It features radiometric, geometric, and atmospheric corrections, as well as orthorectification. AROSICS (An Automated and Robust Open-Source Image Co-Registration Software for Multi-Sensor Satellite Data, [41,42]) is used to detect and correct spatial misregistrations. Atmospheric correction is performed with SICOR (Sensor Independent Atmospheric Correction of optical Earth observation data from multi- and hyper-spectral instruments, [43]) over land surfaces and with Polymer over water surfaces. The module ACwater is embedded in the EnPT suite, which contains the specifications and technical framework required to apply the Polymer algorithm to EnMAP data. A critical part of the implementation is the application of the Polymer algorithm to the two separate EnMAP detectors (VNIR and SWIR) in sensor geometry, as they have a spatial shift of around 20 pixels along-track and 1 pixel across-track, depending on the image position.

**Figure 9:**
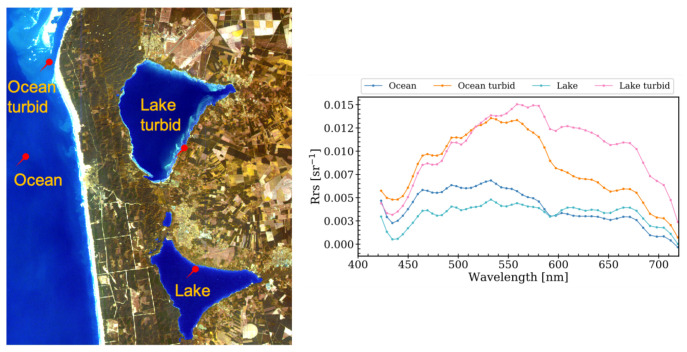
True-color image composite of the simulated EnMAP level 1B data [46] at lake Cazaux et de Sanguinet, located south of Arcachon, France, on 8 November 2017. The spectral signature of four different water types after Polymer was applied to the level 1B data in sensor geometry. The spectra were smoothed using a Gaussian filter with standard deviation of 1.

The ACwater module for running Polymer within EnPT is publicly available, along with its documentation and installation instructions, at [44]. A first application of Polymer to simulated EnMAP data is presented in Figure 9, which shows that Polymer can provide realistic atmospheric correction over water. The Rrs values are in the same range of those observed at the different AERONET-OC sites and Pensacola Bay (Figure 2). Spectra of moderate turbid (pink and orange lines) and clear waters (blue lines) can be distinguished by the increased Rrs in the red bands, characteristic of waters with higher sediment loads [45].

We used HICO data to support algorithm testing in preparation for the EnMAP mission. Compared with HICO, the EnMAP sensor characteristics can potentially lead to less uncertainties of the atmospherically corrected products. For instance, while HICO relied on an SNR of >200:1 (400–600 nm, [23]), EnMAP will have >400:1 (VNIR, [13]) and may improve retrievals over dark inland and coastal waters rich in organic matter. Other advantages are the increased spectral range, covering the SWIR region that can potentially improve the performance of atmospheric correction algorithms over extremely turbid waters [47,48], higher number of bands, and tilt capability ($5^{\circ }$–$30^{\circ }$ from nadir), enabling it to avoid sun glint and improving its revisit time.

## 4. Conclusions

Exploring the fine spectral resolution of hyperspectral sensors can help to improve current water colour remote sensing retrievals, going beyond standard algorithms/retrievals (e.g., band ratio/chlorophyll-a concentration), for such applications as the distinction of different phytoplankton functional groups and water optical constituents. However, the quality of hyperspectral radiometric retrievals depends, in part, on successful AC. In this scope, we presented the first evaluation of hyperspectral radiometric satellite retrievals from Polymer AC, based on HICO data collected between 2010 and 2014 at seven study sites. Polymer HICO retrievals were validated against in situ multi- and hyper-spectral data. The performance statistics of the Polymer AC showed good agreement with in situ measurements. In the green spectral region, the uncertainties can be as low as 16% while, in the red region, they were as high as 66%, in agreement with the range of uncertainties reported in the literature for Polymer applied to multispectral satellite data. Furthermore, Polymer outperformed L2gen, exhibiting lower uncertainties, higher spectral similarity compared with in situ multi- and hyper-spectral measurements, and greater coverage. These findings encourage the application, further development, and validation of Polymer, regarding the new generation of hyperspectral satellite sensors.

## Figures and Tables

**Table 1:** AERONET-OC sites and corresponding data used in this study.

Station Name	Location	Years	Spectral Bands (nm)
COVE_SEAPRISM	$36^{\circ }$ N, $75^{\circ }$ W	2013, 2014	413, 441, 489, 530, 551, 668
MVCO	$41^{\circ }$ N, $70^{\circ }$ W	2010, 2013, 2014	412, 442, 490, 530, 551, 668
LISCO	$43^{\circ }$ N, $70^{\circ }$ W	2010, 2011, 2012, 2014	413, 442, 491, 551, 668
WaveCIS_site_CSI_6	$28^{\circ }$ N, $90^{\circ }$ W	2010, 2011, 2012, 2013, 2014	411, 442, 491, 551, 668
Gloria	$44^{\circ }$ N, $29^{\circ }$ E	2012, 2014	411, 412, 442, 490, 491, 530, 551, 667, 668
Venice	$45^{\circ }$ N, $12^{\circ }$ E	2010, 2011, 2012, 2013, 2014	412, 441, 442, 488, 490, 491, 530, 551, 554, 555, 667, 668

## Data Availability

Not applicable.

## Abbreviations

The following abbreviations are used in this manuscript:

AC	atmospheric correction
AERONET	AErosol RObotic NETwork
AROSICS	An Automated and Robust Open-Source Image Co-Registration Software for Multi-Sensor Satellite Data
BRDF	Bidirectional Reflectance Distribution Function
CHIME	Copernicus Hyperspectral Imaging Mission for the Environment
CHRIS	Compact High Resolution Imaging Spectrometer
DESIS	DLR Earth Sensing Imaging Spectrometer
DLR	Deutsche Zentrum für Luft- und Raumfahrt (German Aerospace Center)
EnMAP	Environmental Mapping and Analysis Program
EnPT	EnMAP processing tool
ESA	European Space Agency
GOME	Global Ozone Monitoring Experiment
HISUI	Hyperspectral Imager Suite
HICO	Hyperspectral Imager for the Coastal Ocean
ISS	International Space Station
MAPD	mean absolute percentage difference
MPD	mean percentage difference
NASA	National Aeronautics and Space Administration
NIR	near-infrared
OC	Ocean Colour
OCI	Ocean Colour Instrument
OLCI	Ocean and Land Colour Instrument
OMI	Ozone Monitoring Instrument
PACE	Plankton, Aerosol, Cloud, ocean Ecosystem
PRISMA	PRecursore IperSpettrale della Missione Applicativa
PRISMA SG	PRISMA Second Generation
SA	spectral angle
SBG	Surface Biology and Geology
SCIAMACHY	Scanning Imaging Absorption Spectrometer for Atmospheric Chartography
Sentinel-5P	Sentinel-5 Precursor
SICOR	Sensor-Independent Atmospheric Correction of optical Earth observation data from multi- and hyper-spectral instruments
SHALOM	Spaceborne Hyperspectral Applicative Land and Ocean Mission
SNR	signal-to-noise ratio
SWIR	short-wavelength infrared
VIS	visible
VNIR	visible and near-infrared
TROPOMI	TROPOspheric Monitoring Instrument
